# Excited state tracking during the relaxation of coordination compounds

**DOI:** 10.1002/jcc.25800

**Published:** 2019-02-23

**Authors:** Juan Sanz García, Martial Boggio‐Pasqua, Ilaria Ciofini, Marco Campetella

**Affiliations:** ^1^ Chimie ParisTech PSL Research University, CNRS, Institute of Chemistry for Life and Health Sciences (i‐CLeHS), FRE 2027 F‐75005 Paris France; ^2^ Laboratoire de Chimie et Physique Quantiques IRSAMC, CNRS et Université Toulouse 3 118 route de Narbonne, 31062 Toulouse France

**Keywords:** state tracking, geometry optimization, TD‐DFT, NTO, overlap

## Abstract

The ability to locate minima on electronic excited states (ESs) potential energy surfaces both in the case of bright and dark states is crucial for a full understanding of photochemical reactions. This task has become a standard practice for small‐ to medium‐sized organic chromophores thanks to the constant developments in the field of computational photochemistry. However, this remains a very challenging effort when it comes to the optimization of ESs of transition metal complexes (TMCs), not only due to the presence of several electronic ESs close in energy, but also due to the complex nature of the ESs involved. In this article, we present a simple yet powerful method to follow an ES of interest during a structural optimization in the case of TMCs, based on the use of a compact hole‐particle representation of the electronic transition, namely the natural transition orbitals (NTOs). State tracking using NTOs is unambiguously accomplished by computing the mono‐electronic wave function overlap between consecutive steps of the optimization. Here, we demonstrate that this simple but robust procedure works not only in the case of the cytosine but also in the case of the ES optimization of a ruthenium nitrosyl complex which is very problematic with standard approaches. © 2019 The Authors. *Journal of Computational Chemistry* published by Wiley Periodicals, Inc.

## Graphical abstract

Exploring potential energy surfaces of excited states (ESs) is necessary for a full understanding of photochemical reactions. To tackle this issue, a new formalism based on the computation of the overlap between NTOs is presented in this article. This NTO‐based approach proves to be able to follow unambiguously a state of interest during ES optimizations of coordination complexes, even in cases that are problematic for standard methods.

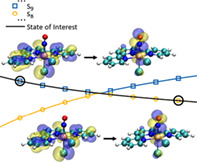

## Introduction

Computational photochemistry has gained an ever growing interest among the scientific community over the last few decades. The plethora of technological applications^1^, ^2^, ^3^, ^4^, ^5^, ^6^, ^7^, ^8^ along with the development of more and more sophisticated spectroscopic techniques has motivated the synergy with theory, permitting a better understanding of photochemical processes at the molecular level. Actually, theoretical photochemistry allows a more thorough interpretation than the traditional electronic analysis of vertical excitations at the Franck–Condon (FC) geometry.^9^, ^10^, ^11^, ^12^, ^13^, ^14^, ^15^, ^16^ It is possible to gain some insights in photochemical mechanisms by characterizing the photochemical pathways which describe the transformation from the reactants to the photoproducts going through all the potential energy surfaces (PESs) involved in the reaction. A full description of this photochemical pathway requires the characterization of the different stationary points along the PESs. In this context, geometry optimizations are of crucial importance for the characterization of these stationary points describing the PES of the different electronic states involved. Unfortunately, geometry optimizations of electronic excited states (ESs) are anything but trivial. Indeed, two main difficulties are faced when optimizing ESs: (1) the inevitable evolution of the electronic rearrangement coupled with the structural reorganization of the system, and (2) the possible state crossings during the optimization procedure. Both problems are related; taking large steps in geometry optimizations certainly results in large electronic rearrangements for all ESs which may harden the task of identifying the ES of interest. Besides, the order of ESs is more likely to change when large optimization steps are taken. Performing ES geometry optimizations ignoring these risks may rapidly lead to crossing states of different electronic nature during the procedure, losing irrevocably the state of interest. In order to address the first issue, one can simply use a small step size in the optimization algorithm. On the other hand, to overcome the second problem, standard state‐tracking procedures are implemented in general quantum chemistry packages such as FIREFLY^17^, ^18^ (formerly PC‐GAMESS) or GAUSSIAN.^19^ One of these unequivocal standard state‐tracking procedures is based on the analysis of the overlap between the reference ES and all the ESs computed at each step using the configuration interaction (CI) vectors. A more original idea, implemented few years ago in Q‐CHEM,^20^ consists in computing the overlap between the attachment–detachment electronic transition densities.^21^ The wave function (density) overlap measures how similar the compared wave functions (densities) are. In this article, we propose yet another formalism to compare different electronic ESs. Unlike the methods mentioned afore, this new formalism is based on the overlap between the natural transition orbitals (NTOs) describing the ESs. This new formalism proves to be very efficient in the problematic ES optimizations of transition metal complexes (TMCs).

A detailed computational study of the photochemical pathways involving TMCs is very challenging due not only to the high density of electronic states involved in these systems, but also to their complex electronic structure. Unsurprisingly, not so many computational studies can be found regarding the mechanistic description of photochemical reactions involving directly the metal center^22^, ^23^, ^24^, ^25^, ^26^, ^27^, ^28^, ^29^, ^30^, ^31^, ^32^ (compared to the available literature concerning organic systems, see Refs.^9^, ^11^, ^33^, ^34^, ^35^, ^36^, ^37^, ^38^, ^39^ for relevant review articles). One of the main limitations of the TMC photochemical studies is the difficulty of optimizing an *n*th ES. For instance, if we are interested in optimizing the absorbing bright state and it is not one of the lowest ESs, the optimization of that bright state becomes extremely difficult due to the high density of states which results in frequent crossings between states of different electronic nature during the geometry optimization. Needless to say, this is also true for any kind of ES. One of the cheapest (and probably affordable) computational methods to optimize ESs of TMC is the time‐dependent density functional theory (TD‐DFT). In contrast with organic chromophores where the TD‐DFT ES optimization has become a standard practice,^15^, ^16^, ^40^ TMC ES optimizations using TD‐DFT are still very difficult to perform. Indeed, in previous TMC photochemical studies,^26^, ^27^ we have not been able to perform successfully TD‐DFT ES optimizations. However, in order to gain a deeper understanding on the photochemical mechanisms, it would be necessary to understand the role of the higher ESs. This lack of information has motivated the development of the new formalism presented in this article to compare different ES of TMC (or any other chemical system). This formalism has been implemented in an external tool which interfaces with the GAUSSIAN quantum package so that TMC ES optimizations can be performed. Although the overlap^41^, ^42^, ^43^, ^44^, ^45^ and the NTOs^46^ are used in many other quantum chemistry applications, to the best of our knowledge, this approach addressing the ES optimization issue by combining these simple concepts has never been tested to date.

This article is organized as follows: In Theoretical Background section, a general overview of the new method and a detailed description of the algorithm behind the external tool developed are given. In Computational Details section, all technical parameters used for each calculation are specified. Next, in Cytosine section, ES optimizations obtained for the cytosine are discussed, while in *cis*‐(Cl,Cl)[RuCl_2_(NO)(tpy)]^+^ complex section, the results obtained for the ruthenium nitrosyl ES optimizations are analyzed. Finally, a summary of the results and some general conclusions are drawn in the last section.

## Theoretical Background

In order to perform ES tracking optimizations an in‐house code named “**S**teepest **D**escent minimization using **N**atural **T**ransition **O**rbitals” (SDNTO) has been developed. The code is written in Fortran90 and acts as an external tool, perfectly interfaced with the GAUSSIAN quantum package. Thanks to this program, it is possible to perform an ES minimization by means of a steepest descent algorithm,^47^ following an ES characterized by a specific electronic arrangement (diabatic state). For convenience, this state will be considered as the reference state (RS). The SDNTO code calls GAUSSIAN at every step to perform a vertical ES calculation in order to obtain the excitation energies and the coefficients of the NTOs^46^ for all of the considered transitions. In addition, it requires the calculation of the gradient of the ES of interest. At each step *n*+1, SDNTO computes the overlap between the NTOs of the RS (computed at the previous step *n*) and the NTOs of all the other ESs (computed at step *n*+1). The overlap function (*S*_NTO_) has been defined as follows:

(1) $$S_{\mathrm{NTO}}=\sum_{i=1}^{N}c_{i}^{n}\left|\int d^{3}r\;\phi_{i,\mathrm{RS}}^{n}\left(x^{n};r\right)\;\phi_{i,\mathrm{RS}}^{n+1}\left(x^{n+1};r\right)\right|$$

or

(2) $$S_{\mathrm{NTO}}=\sum_{i=1}^{N}c_{i}^{n}\int d^{3}r\left|\phi_{i,\mathrm{RS}}^{n}\left(x^{n};r\right)\right|\left|\phi_{i,\mathrm{RS}}^{n+1}\left(x^{n+1};r\right)\right|$$

**Figure 1:**
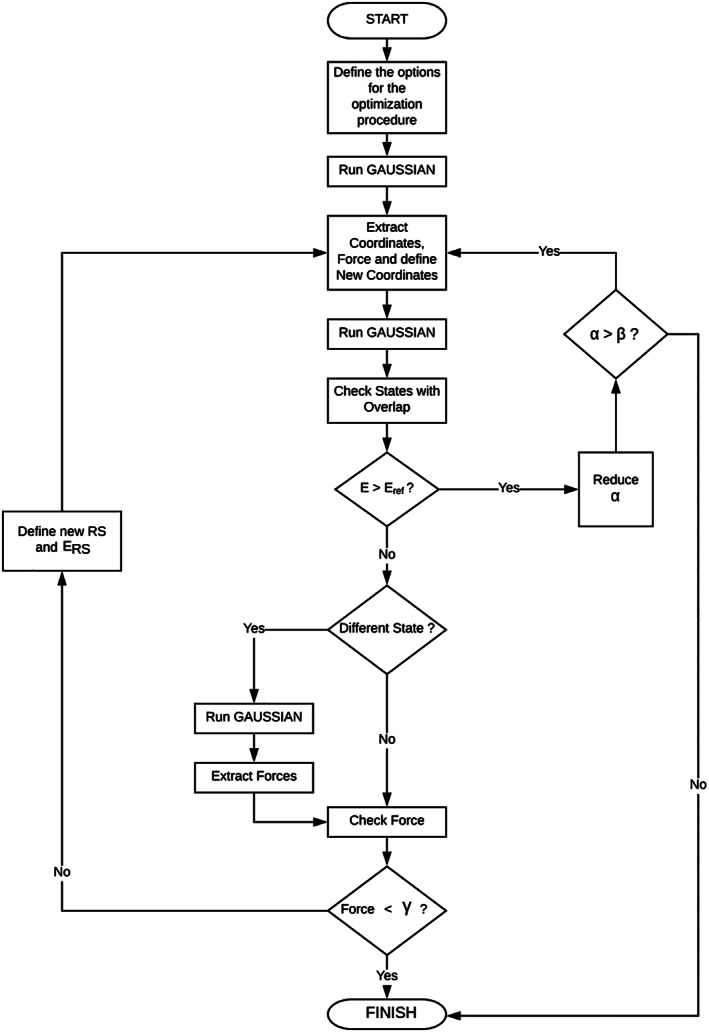
Flowchart of the SDNTO program.

In eqs. (1) and (2), *i* goes from 1 to the number of orbitals *N* taken into account, namely the number of NTOs with an eigenvalue greater than a given threshold. $c_{i}^{n}$ are the eigenvalues of the NTOs related to the RS, *i.e*. the occupation numbers, computed at step *n*.$\;\;\phi_{i,\mathrm{RS}}^{n}\left(x^{n};r\right)$ represents the NTO *i* computed at step *n* for the RS, with ***x***^*n*^ representing the molecular geometry at the optimization step *n*. The *S*_NTO_ has been defined as the pure overlap between pairs of NTOs weighted by the populations of the RS (holes and particles are analyzed independently). The orbitals, whose eigenvalue is below a given threshold, are neglected. By virtue of the overlap function *S*_NTO_, it is possible to keep track of an ES with the same electronic nature along the whole minimization procedure. It is worth noticing that two different types of overlap *S*_NTO_ are available in the SDNTO code: the module of the integral eq. (1) and the integral of the module eq. (2). Both formulations avoid possible problems with the phase alignment of the wave functions involved. Furthermore, with the SDNTO program it is possible to compute the overlap using either two different molecular geometries (step *n* and step *n*+1) or only the geometry of step *n*. In the latter case, all NTOs are centered on the “initial” geometry of the step *n*, that is, imposing ***x***^*n*+1^ to be equal to ***x***^*n*^. In the former case, the corresponding geometries computed at step *n* and *n*+1 are used. The flowchart describing the algorithm implemented in the SDNTO code is represented in Figure 1. The basic algorithm is composed as it follows:

**Step 1**: The initial GAUSSIAN input file is given as an input to the SDNTO program. All options for a vertical TD‐DFT calculation are directly read from this initial input file, and subsequently used at each step of the SDNTO optimization. The RS is initially read with the rest of the options and it is updated at each step.

**Step 2**: At each optimization step *n*, a vertical TD‐DFT calculation is performed in order to recover the energy ($E_{\mathrm{RS}}^{n}$), gradient ($\nabla E_{\mathrm{RS}}^{n}$) and the NTOs of the RS.

**Step 3**: New coordinates for the next optimization step *n*+1 are generated by using a steepest descent algorithm. The new coordinates are thus computed as:

(3) $$x^{n+1}=x^{n}-\alpha \cdot \nabla E_{\mathrm{RS}}^{n}$$

where ***x***^*n*^ is a vector containing all the coordinates (expressed in Bohr) of the system at step *n*, *α* is a dimensional constant (the units are Bohr^2^/Hartree) whose value can be modified by the user, $\nabla E_{\mathrm{RS}}^{n}$ is the energy gradient (in Cartesian coordinates) of the RS at the optimization step *n* and ***x***^*n*+1^ represents the new coordinates to be used in the optimization step *n*+1.

**Step 4**: At step *n*+1, a vertical TD‐DFT calculation is carried out to retrieve all the NTOs of all the ESs computed.

**Step 5**: Then, the overlap between the RS NTOs (of step *n*) and the NTOs of all the ESs computed at the optimization step *n*+1 is determined. The state with the larger *S*_NTO_ value becomes the new RS. Clearly, a sufficient number of states should be kept during the optimization in order to allow a significant matching.

**Step 6**: The SDNTO program checks whether the energy of the RS at step *n*+1 is greater than the one computed at step *n*. If so, *α* is halved. If the new *α* value is lower than a given threshold (β) the procedure stops, otherwise the program restarts the loop from step 3.

**Step 7**: Once the energy has decreased, if the position of the RS among all the ESs at the optimization step *n*+1 is different than the one at optimization step *n*, then a supplementary SP TD‐DFT calculation needs to be done in order to compute the energy gradient $\nabla E_{\mathrm{RS}}^{n}$ of the correct RS at the new geometry ***x***^*n*+1^.

**Step 8**: The SDNTO program checks whether the maximum gradient associated to the new RS is lower than a given threshold (γ). If so, the procedure stops and the optimization can be considered as finished, otherwise it restarts the loop from step 3.

Overall, the SDNTO code computes, at each step, the overlap among the NTOs of the RS (computed in the previous step *n*) and the NTOs of all the other ESs (computed at step *n*+1). The state with the greater overlap will be the new RS. Once the new RS has been found, its gradient is computed. Finally, the new coordinates at step *n*+1 are generated following the steepest descent of the gradient. A constant *α* that multiplies the force is used in order to control the step size.

## Computational Details

All electronic structure calculations were performed in the gas phase with the GAUSSIAN quantum package.^19^ DFT and TD‐DFT were used to compute the ground and the ESs of each molecule, respectively. The ground state (GS) geometry of the cytosine molecule was optimized as a starting point for the ES TD‐DFT optimizations. The preliminary GS DFT and the ES TD‐DFT optimizations were performed using the same hybrid functional PBE0^48^ with the (6–31+G[d])^49^ diffuse‐augmented polarization valence‐double‐*ζ* basis set with one set of *d* polarization functions^50^, ^51^ and a set of *s* and *p* diffuse functions^52^, ^53^ for all atoms but hydrogens. Starting from a previously optimized structure in acetonitrile,^27^ the GS geometry of the *cis*‐(Cl,Cl)[RuCl_2_(NO)(tpy)]^+^ complex was re‐optimized in vacuum using the standard hybrid functional B3LYP,^54^, ^55^, ^56^ as in Ref. 27 with a double‐*ζ* Ahlrichs‐type basis set for the hydrogen atoms, a triple‐*ζ* Ahlrichs‐type basis set with one set of *d* polarization functions for the second‐ and third‐row elements,^57^ and a Stuttgart relativistic effective core potential^58^ (including 28 core electrons) with its associated basis set^58^ including two sets of *f* functions for the ruthenium,^59^ this basis set will be denoted hereafter as “BS1.” This optimized GS geometry was used as a starting point for the ES TD‐DFT optimizations. The same hybrid functional BHandHLYP^54^, ^56^, ^60^ used in Ref. 27 was employed to perform all the ES TD‐DFT optimizations for the *cis*‐(Cl,Cl)[RuCl_2_(NO)(tpy)]^+^ complex. These optimizations were carried out using a polarization valence‐double‐*ζ* (6‐31G[d])^49^ basis set with one set of *d* polarization functions^50^, ^51^ for all atoms but hydrogens, a set of *s* and *p* diffuse functions^52^, ^53^ for the chlorine atoms and the double‐*ζ* quality LANL2DZ^61^ basis set with its associated effective core potential^62^ (including 28 core electrons) for the ruthenium atom, this basis set will be denoted hereafter as “BS2.” The BS2 basis set has proven to be good enough to recover the main character of the brightest state, within the range from 300 to 500 nm, reported in Ref. 27 and yield a similar excitation energy (302.5 nm). At the end of all the ES geometry optimizations performed with GAUSSIAN or with SDNTO, vibrational frequency analysis were performed at the same level of theory, in order to verify the nature of the stationary points. Cartesian coordinates and energies of all the optimized stationary points can be found in Tables S1–S7, Supporting Information.

In the ES optimizations performed with the SDNTO program the convergence threshold for the maximum gradient (γ) and the threshold for the NTOs selection have been set to 4.5 × 10^−4^ Hartrees/Bohr and 0.3, respectively. These parameters have been used for both systems, the cytosine molecule and the *cis*‐(Cl,Cl)[RuCl_2_(NO)(tpy)]^+^ complex. In all calculations, eq. (1) was used to compute the *S*_NTO_. Likewise, the steepest descent algorithm as implemented in the GAUSSIAN quantum package has been used for all the ES optimizations performed with this program. The maximum size for the steepest descent steps has been set to 0.01 Bohr. For all these GAUSSIAN ES optimizations, the maximum gradient and the threshold for the maximum displacement have been set to 4.5 × 10^−4^ Hartrees/Bohr and 1.8 × 10^−3^ Bohr, respectively.

## Results and Discussion

**Figure 2:**
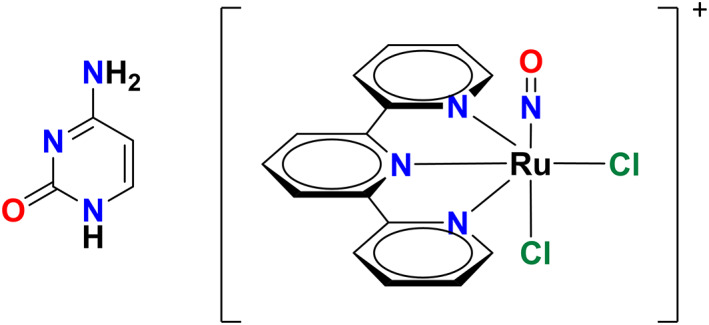
Structures of the cytosine 1H‐amino‐keto tautomer (left) and the *cis*‐(Cl,Cl)[RuCl_2_(NO)(tpy)]^+^ complex (right). [Color figure can be viewed at wileyonlinelibrary.com]

In order to prove the reliability of the SDNTO approach a small organic chromophore, the cytosine molecule, (Fig. 2), has been used as a case study. The cytosine is particularly interesting because it presents, very close to the FC region, a sloped conical intersection between the first two singlet ESs S_1_ and S_2_. Starting from the FC geometry, the optimizations of states S_1_ and/or S_2_ are unlikely to finish at the same starting electronic ES, unless a state‐tracking algorithm is used. In this study case, for the optimizations of the first two singlet ESs, both algorithms afford virtually the same results (Table 1). Additionally, we have chosen a remarkably difficult case of TMC, the ruthenium nitrosyl family of complexes, in which, so far, we have not been able to optimize the higher ESs of interest, namely the brightest state within the spectral range from 300 to 500 nm.^26^, ^27^, ^28^ A modestly sized ruthenium nitrosyl complex has been used for the study: the *cis*‐(Cl,Cl)[RuCl_2_(NO)(tpy)]^+^ complex from Ref. 27 (Fig. 2). The successful optimization of the ninth singlet ES of this complex illustrates the robustness of our formalism.

**Table 1:** Comparison of the energy and structural parameters obtained using the standard optimization algorithm (Std.) and SDNTO. Refer to the text for the nomenclature.

	ΔE (SDNTO‐Std.)/kcal mol^−1^	RMSD (SDNTO‐Std.)/Å
*π* → π^*^	−0.109	0.008
*n* → π^*^	−0.393	0.049

### Cytosine

The cytosine molecule, being one of the nucleic acid bases is of extreme biological relevance. It is a derivative of the pyrimidine, composed by a heterocyclic aromatic ring and two functional groups: an amine group attached in position 4 and a carbonyl in position 2 (see Fig. 2). Although six different isomers exist, we have focused our attention only on the so‐called 1H‐amino‐keto tautomer, whose molecular structure is reported in Figure 2. Many theoretical and experimental studies have been carried out on this molecule analyzing its optical properties.^63^, ^64^, ^65^, ^66^, ^67^, ^68^ According to these studies, the first two ESs of the cytosine are: a *π* → π^*^(S_1_) and an *n* → π^*^ (S_2_) transition, the former is a bright state, while the latter, involving the carbonyl oxygen, is a dark one. In the well‐established decay mechanism, an internal conversion (IC) occurs from the optically excited *π* → π^*^ to the dark *n* → π^*^ state. Taking this mechanism into account, we have performed a state‐tracking geometry optimization of both ESs, using both the standard approach implemented in the GAUSSIAN quantum package and the SDNTO code.

**Figure 3:**
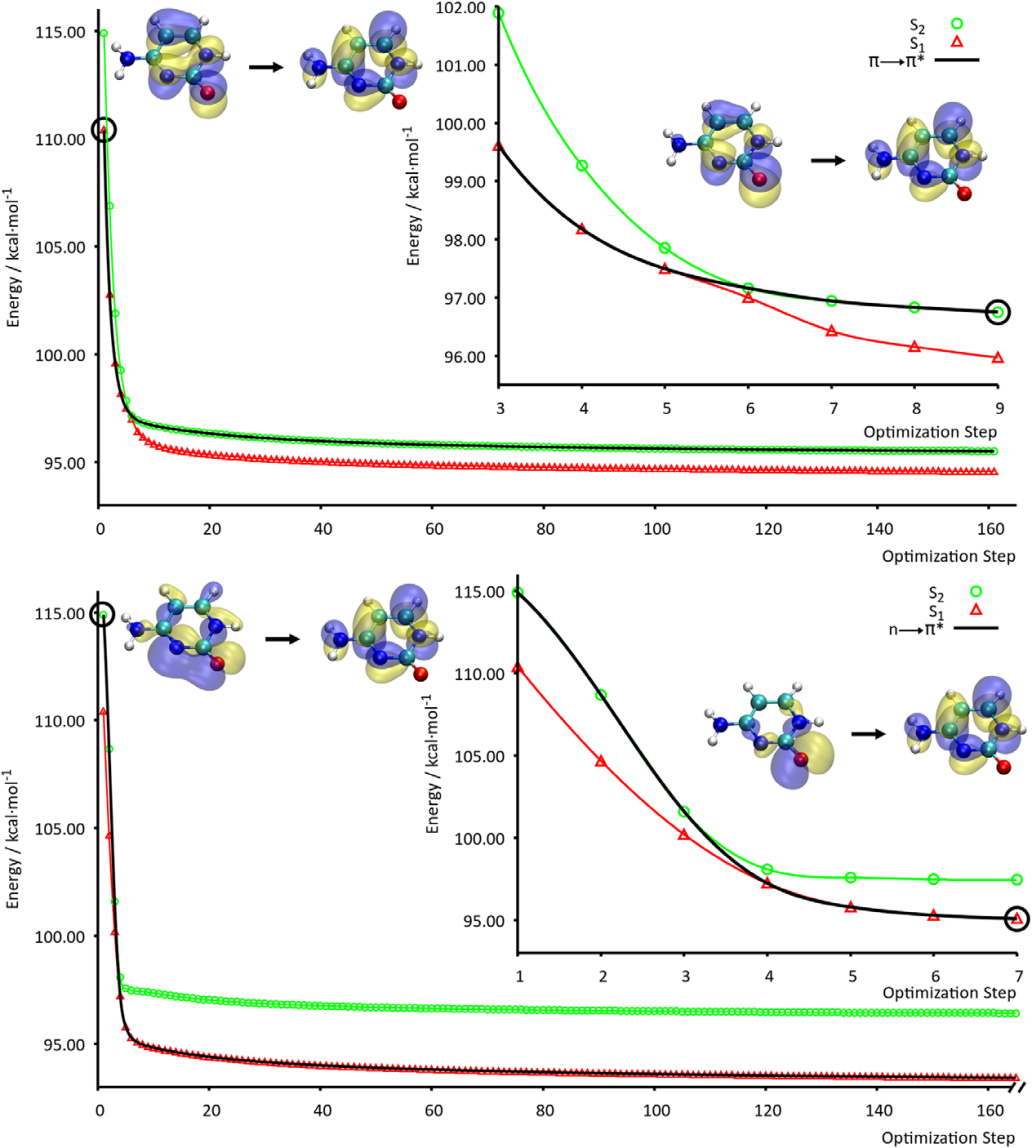
Evolution of the relative energy (with respect to the energy of the GS minimum) during the minimization of the *π* → π^*^ state (upper panel) and the *n* → π^*^ state (lower panel). In red and green are represented the energy of the first and second ES, respectively, along the minimization procedure. The black line represents the RS energy at each minimization step. RS NTOs at the FC region and after the crossing point (black circles) are depicted. [Color figure can be viewed at wileyonlinelibrary.com]

First, we discuss the optimization of the *π* → π^*^ state as it is the RS for the first optimization. In Figure 3, the relative energies of the first two ESs (green circles and red triangles) and the energy of the RS (black line), computed along the minimization procedure, are reported. The NTOs computed at the FC geometry and after the S_1_/S_2_ crossing point are also depicted in Figure 3. For this transition, we have reported only the NTOs that have a contribution greater than 0.3. This particular transition is characterized by a single pair of hole‐particle orbitals. Obviously, at the beginning of the minimization (first step), the RS is the first ES. It remains the first state up to the fifth step, at the next point SDNTO changes the RS: it becomes the second transition (see the black line of Fig. 3). If we take a look at the NTOs of the second ES, computed at the ninth step (few steps further from the crossing point), we can see that the program correctly follows the same electronic state, since the transition is always characterized by the same pair of *π* → π^*^ orbitals. From here on, the *π* → π^*^ state becomes higher in energy compared to the *n* → π^*^ one: the ES ordering changes. Once the crossing has taken place, the *π* → π^*^ transition remains the second one up to the end of the minimization.

Second, we analyze the optimization of the *n* → π^*^ state. Even in this case, SDNTO is able to track the right state during the optimization procedure (see Fig. 3). In the FC region, the *n* → π^*^ state is the second one, as expected, but from the third to the fourth step, there is a crossing point and it becomes the first ES. It can be clearly seen by simple inspection of the hole‐particle NTOs depicted in Figure 3. As for the *π* → π^*^ state, after the initial crossing point, there are no other crossing points during the optimization. After the frequencies calculation, it has been verified that the last geometry corresponds to a stationary point, being a transition state (see Supporting Information for further details). It is worth noticing that an optimization procedure may lead to any kind of stationary point.

For both of these minimization procedures, *α* has been fixed at 0.4 Bohr^2^/Hartree. For the optimization of the *π* → π^*^state, 160 steps were needed. In the case of the *n* → π^*^ optimization, 174 were initially needed and *ca*. 150 additional steps (increasing gradually the *α* value) were needed in order to make the calculation converge to the stationary point found. In order to verify that our algorithm works properly, the same optimizations were performed with the standard algorithm implemented in GAUSSIAN, providing virtually the same results (see Table 1, and Fig. S1, Supporting Information). Thus, we are confident that our algorithm is reliable and performs an ES steepest descent optimization following the right state. Indeed, as reported in Table 1, the energy difference between the optimized ESs obtained with the two methods is 0.1 kcal/mol for the *π* → π^*^ state and 0.4 kcal/mol for the other one. The corresponding root‐mean‐square displacements (RMSDs), computed on the superposed minimum geometries are very low accordingly. All these results emphasize the fact that both procedures lead to similar geometries. Finally, we have computed the energy difference between the optimized geometries of the *n* → π^*^ state and the S_1_/S_2_ crossing point. Using the NTO tracking‐based approach, this energy is 4.1 kcal/mol, whereas with the standard procedure is 5.0 kcal/mol. The ensuing energies are comparable to those reported in the literature (3.6 kcal/mol).^67^ All geometries are reported in Supporting Information.

### *cis*‐(Cl,Cl)[RuCl_2_(NO)(tpy)]^+^ complex

Ruthenium nitrosyl complexes are of paramount interest among the scientific community due to their capability of undergoing either photorelease^69^, ^70^, ^71^, ^72^ or photoisomerization^2^, ^73^, ^74^, ^75^ reactions. DFT calculations have proven to be suitable for the characterization of the main PESs involved in these photoinduced processes. Thanks to these calculations, the rationalization of these photoinduced chemical reactions was possible.^26^, ^27^, ^28^ The photoisomerization mechanism was also confirmed by ultraviolet–visible absorption spectroscopy^76^ and quite recently by MS‐CASPT2 calculations.^31^ This latter study shed some light on the importance of the higher ESs. However, in all the theoretical studies that have been conducted so far, many assumptions have been made, due to the limitations of the computational approaches used.

In the computational studies involving a ruthenium center, the main assumption is that, after light absorption, the singlet ESs, initially populated, rapidly deactivate to the lowest triplet state by nonradiative decays (intersystem crossing (ISC) and IC).^22^, ^23^, ^24^, ^25^, ^26^, ^27^, ^28^, ^29^, ^30^, ^31^ This assumption is supported by theoretical^32^, ^77^ and experimental^78^, ^79^, ^80^, ^81^, ^82^, ^83^ studies. Then, the photochemical pathway is computed on the lowest triplet PES. To account for possible nonradiative decay back to the singlet GS from the lowest triplet state by ISC, singlet/triplet crossings are determined as they provide efficient funnels for deactivation. The optimization of the higher ESs will provide additional information about the mechanism of these photochemical reactions.

**Figure 4:**
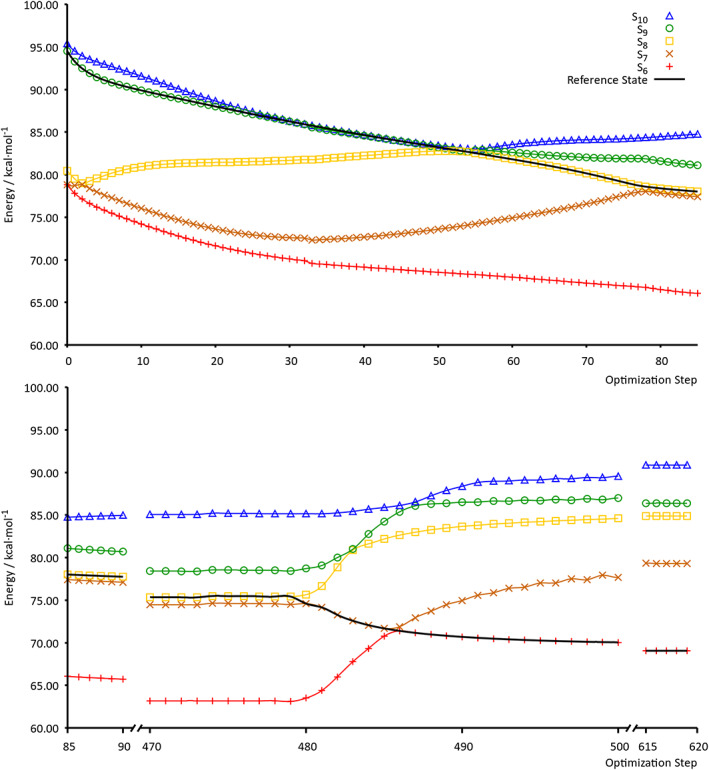
Evolution of the relative energy (with respect to the energy of the GS minimum) of the *cis*‐(Cl,Cl)[RuCl_2_(NO)(tpy)]^+^ ESs from the 6th to 10th along the minimization procedure are reported. The RS is represented by a continuous black line. The first 469 steps have been computed using *α* = 0.2 Bohr^2^/Hartree, while the last 150 steps have been computed using *α* = 0.9 Bohr^2^/Hartree. [Color figure can be viewed at wileyonlinelibrary.com]

The *cis*‐(Cl,Cl)[RuCl_2_(NO)(tpy)]^+^ complex is a perfect model that represents the ruthenium nitrosyl family of complexes. For this molecule, we have chosen to optimize the brightest ES within the spectral range from 300 to 500 nm (tpy → RuNO) which is the ninth one. Only the energies of the ESs involved along the relaxation path of the RS, ranging from the 6th to the 10th, are reported in Figure 4. It can clearly be seen that following the right RS is much more complicated compared to the cytosine optimization. In this case, there are more crossing points, and the number of states involved during the minimization is greater, namely five, as depicted in Figure 4. Starting from the ninth ES, the RS changes up to become the sixth. To ensure that the minimization has been performed correctly, that is, the RS is followed properly, we have printed the hole and particle NTOs at each step for the RS. We have created two animated videos, using the NTO images at each optimization step, one for the hole (SI, ru_complex_SDNTO_hole.avi) and the other one for the particle (SI, ru_complex_SDNTO_particle.avi). These videos clearly show that during the minimization the nitrosyl group tilts down, and the NTOs change accordingly. Furthermore, the movies show that the electronic rearrangement of both the hole and the particle change progressively according to the geometry changes. These results ensure that we are following the right RS.

In order to be sure that the first problem described in the introduction was avoided small steps were taking during the ES optimizations (controlled by the maximum step size used in the standard algorithm set to 0.01 Bohr and by the *α* constant set to 0.2 Bohr^2^/Hartree used by the SDNTO program) (see Table S8, Supporting Information for further details). Although the main concern of this work is not improving the convergence of the optimization procedure, but to provide a robust state‐tracking algorithm, further analysis to test whether the optimization convergence can be improved have been performed. Indeed, increasing the step size during the ES optimizations (using *α* = 0.8 and 0.9 Bohr^2^/Hartree with the SDNTO program), the procedure still works perfectly fine, yielding similar results and always following the right diabatic state (see Table S9, Supporting Information). These results suggest that in the optimization procedure the convergence can be greatly improved, probably with second‐order methods (such as Quasi Newton–Raphson algorithms) and that the NTO state‐tracking‐based method presented in this study may also work coupled with molecular dynamics simulations.

**Figure 5:**
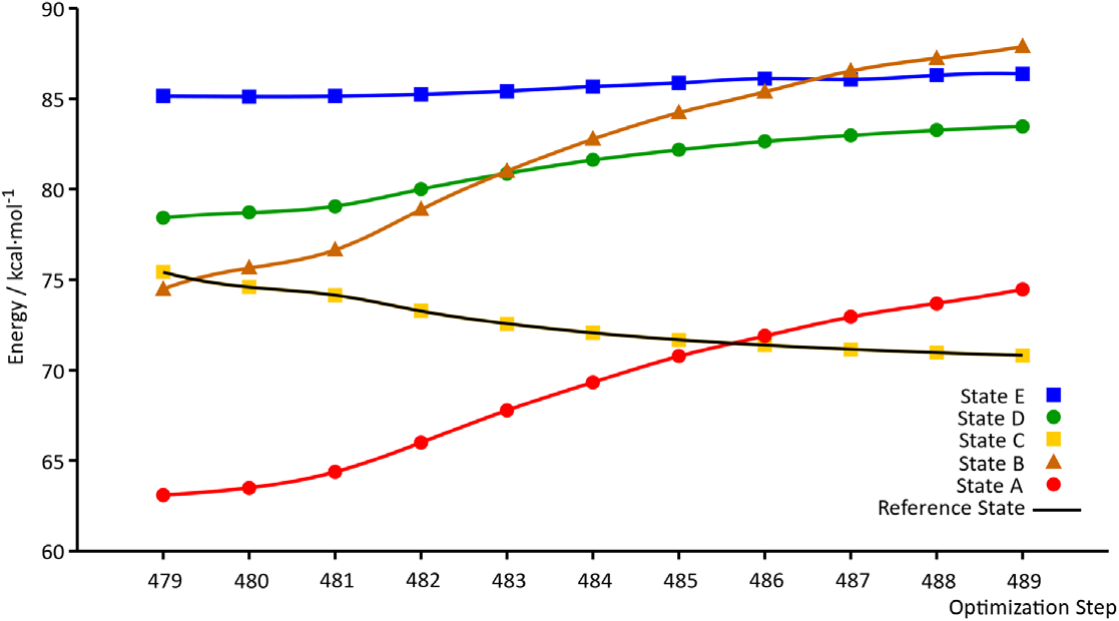
Evolution of the relative energy (with respect to the energy of the GS minimum) of the *cis*‐(Cl,Cl)[RuCl_2_(NO)(tpy)]^+^ diabatic ESs along the minimization procedure. SDNTO optimization from step 479 to step 489. [Color figure can be viewed at wileyonlinelibrary.com]

It is worthwhile to mention that we were unable to follow this state using standard optimization approaches (even with a small step size, i.e., 0.01 Bohr), the state of interest changing abruptly during the optimization procedure (see Supporting Information, ru_complex_std_hole.avi and ru_complex_std_particle.avi). Additionally, the new formalism has been tested using the same steps of the standard state‐tracking optimization, thus strictly the same step sizes. In this case, the NTO's overlap‐based state‐tracking approach proves again to follow always the right state of interest, even where the standard approach fails (see Table S10, Supporting Information for further information). Furthermore, this formalism based on the NTOs overlap is able to follow not only the state of interest during the ES optimization, but also the evolution of all the other diabatic states (with no additional electronic structure calculations) as shown in Figure 5. This analysis reveals how this robust NTOs‐based formalism is able to track unambiguously all different ESs (Tables S11 and S12) from the 479th to the 489th step of the *cis*‐(Cl,Cl)[RuCl_2_(NO)(tpy)]^+^ SDNTO optimization (where many state crossings occur). Indeed, knowing the energy evolution of the different diabatic states along a specific molecular geometry deformation gives further important insights on the mechanism of a photochemical reaction. Once again, the new formalism presented in this work has proven to be an adequate tool for this type of analysis.

## Conclusions

In this work, we have presented a new formalism to track electronic ESs of different nature. This formalism based on the NTOs overlap has been applied to ES geometry optimizations, permitting to follow a specific state along the process. An ES optimization of TMC represents a major obstacle in TD‐DFT calculations. The developed algorithm, which unambiguously follows a specific ES, has proven to solve this major issue. The algorithm has been tested in two different systems: (1) the cytosine (small organic chromophore) for which standard state‐tracking procedures work fine too, and (2) a ruthenium nitrosyl complex for which it is very challenging to optimize ES using the standard procedures. Results obtained with a standard procedure as well as with the formalism presented here are compared for all the systems studied. Regarding the cytosine, we have optimized the first two ESs. The state‐tracking procedures used yield similar results. On the other hand, in the case of the ruthenium nitrosyl complex, only the NTO based formalism presented here is able to follow correctly the ES of interest during the whole geometry optimization. Indeed, as stated by Martin in the original paper,^46^ NTOs provide a compact representation of the electronic transitions, compared to the classical CI representation. This transformation from the canonical orbitals to the NTO representation proves to be essential in order to achieve a successful state‐tracking algorithm.

Our original state‐tracking algorithm provides a valuable tool for tackling TMC photoreactivity, opening a wide scope of possibilities. Using the approach presented here, the role of the initial populated ESs or even the role of the intermediate states involved in photochemical reactions can be elucidated.

## Supporting information


**Appendix S1**: Supplementary Video

**Appendix S2**: Supporting Information
