# Supplementary material for: Excited state tracking during the relaxation of coordination compounds
Source: J Comput Chem. 2019 Feb 23;40(14):1420–8. doi: 10.1002/jcc.25800 (PMC8247441; doi:10.1002/jcc.25800)

Supporting Information for:

**Excited State Tracking during the Relaxation of Metallic Coordination Complexes**

Juan Sanz García^a^* , Martial Boggio-Pasqua^b^ , Ilaria Ciofini^a^ , Marco Campetella^a,c^*

^a^ *Chimie ParisTech, PSL Research University, CNRS, Institut de Recherche de Chimie Paris, F-75005 Paris, France*

^b^ *Laboratoire de Chimie et Physique Quantiques, IRSAMC, CNRS et Université Toulouse 3,* *118 route de Narbonne, 31062 Toulouse, France*

^c^ Current Address:*Sorbonne Université, CNRS, Institut des Nanosciences de Paris, UMR7588, F-75252 Paris, France*

**Table of Contents**

**Table S1.** Cytosine ground state structure and energies 2

**Table S2.** $\pi\to\pi^{\boldsymbol{*}}$Optimized geometry (std. algorithm) and energies 3

**Table S3.** $\pi\to\pi^{\boldsymbol{*}}$Optimized geometry (SDNTO) and energies 4

**Table S4.** $n\to\pi^{\boldsymbol{*}}$Optimized geometry (std. algorithm) and energies 5

**Table S5.** $n\to\pi^{\boldsymbol{*}}$Optimized geometry (SDNTO) and energies 6

**Table S6.** *cis*-(Cl,Cl)[RuCl_2_(NO)(tpy)]^+^ ground state structure and energies 7

**Table S7.** *cis*-(Cl,Cl)[RuCl_2_(NO)(tpy)]^+^ Optimized geometry (SDNTO) and energies 8

**Figure S1.** Superposed optimized structures of the cytosine molecule 9

**Table S8.** Displacements during the *cis*-(Cl,Cl)[RuCl_2_(NO)(tpy)]^+^ SDNTO optimization 10

**Table S9.** Displacements during additional *cis*-(Cl,Cl)[RuCl_2_(NO)(tpy)]^+^ SDNTO optimizations with larger *α* values 12

**Table S10.** Comparing the standard and the NTO-based state-traking approaches 15

**Table S11.** NTOs (hole) of the diabatic states from the *cis*-(Cl,Cl)[RuCl_2_(NO)(tpy)]^+^ SDNTO optimization 16

**Table S12.** NTOs (particle) of the diabatic states from the *cis*-(Cl,Cl)[RuCl_2_(NO)(tpy)]^+^ SDNTO optimization 17

**Table S****1.** Optimized Cartesian coordinates and energies of the cytosine’s ground state at the PBE0/6-31+G(d) level of theory in vacuum.

C 0.773840041436 0.836607374456 -0.115583901760

C 1.445640123445 2.016251467595 -0.106912411604

C 0.639052194787 3.196422188725 0.040708659013

C -1.351083996383 1.998501136361 0.157938740305

H -1.092956167534 -0.040790659260 0.007688280794

H 1.265852779715 -0.125754442735 -0.219975117095

H 2.524598094830 2.055045529490 -0.197162605755

N 1.246256680526 4.407446811166 0.085279030100

H 2.212403375442 4.517271862543 -0.175683013004

H 0.652520100703 5.224019550452 0.078139399573

N -0.671782972452 3.182539062254 0.161094423725

O -2.559476299671 1.875475286131 0.266578755286

N -0.569883954845 0.824514832821 0.010574760222

| **Energies (a.u.)** | | -394.509180 |
| --- | --- | --- |
| *E* | 0.099504 | |
| Zero-point correction | -394.409676 | |
| Sum of electronic and  thermal enthalpies | -394.401977 | |
| Sum of electronic and  thermal free energies | -394.440517 | |

**Table S2.** Optimized Cartesian coordinates and energies of the cytosine’s $\pi\to\pi^{\boldsymbol{*}}$state at the PBE0/6-31+G(d) level of theory in vacuum. Results obtained with a standard optimization algorithm.

C 0.787735940852 0.779648757498 -0.114192646506

C 1.450958801042 2.024995718867 -0.095355023336

C 0.725175030921 3.180097510123 0.059413564799

C -1.252829211539 2.012859712915 0.158273822265

H -1.167019146918 -0.008807403430 -0.001848599111

H 1.256035579909 -0.182490359368 -0.250121364572

H 2.531969064144 2.051254354510 -0.193443066927

N 1.248525465844 4.441238622962 0.163596709165

H 2.181841385035 4.598914822014 -0.187024536262

H 0.590043820360 5.187699192079 -0.014656809811

N -0.681988688666 3.173818398649 0.195282391413

O -2.555837081424 1.882454207093 0.263558113610

N -0.599630959561 0.825866466086 0.009202445075

| **Energies (a.u.)** | | -394.357144 |
| --- | --- | --- |
| *E* | 0.096350 | |
| Zero-point correction | -394.260794 | |
| Sum of electronic and  thermal enthalpies | -394.252706 | |
| Sum of electronic and  thermal free energies | -394.291836 | |

**Table S3.** Optimized Cartesian coordinates and energies of the cytosine’s $\pi\to\pi^{\boldsymbol{*}}$state at the PBE0/6-31+G(d) level of theory in vacuum. Results obtained with SDNTO.

C 0.786885312200 0.781585736000 -0.122329390000

C 1.442629400801 2.029655345599 -0.110364009200

C 0.712994642001 3.183047783200 0.038528419601

C -1.258673785799 2.008302675000 0.150927886600

H -1.161443348601 -0.016268792399 0.015321748600

H 1.263840829399 -0.180095299201 -0.225710809800

H 2.523884872601 2.063763705199 -0.200429561400

N 1.250431167600 4.435528274400 0.144782906603

H 2.195016479399 4.582233054802 -0.177249060000

H 0.614246359198 5.211460058598 0.031117277797

N -0.694272100199 3.174007016200 0.173143340601

O -2.560314133799 1.871659174201 0.264129110200

N -0.600234694800 0.822663268400 0.010807140600

| **Energies (a.u.)** | | -394.356971 |
| --- | --- | --- |
| *E* | 0.096034 | |
| Zero-point correction | -394.260937 | |
| Sum of electronic and  thermal enthalpies | -394.252657 | |
| Sum of electronic and  thermal free energies | -394.292297 | |

**Table S4.** Optimized Cartesian coordinates and energies of the cytosine’s $n\to\pi^{\boldsymbol{*}}$state at the PBE0/6-31+G(d) level of theory in vacuum. Results obtained with a standard optimization algorithm.

C 0.792793000000 0.784570000000 -0.128040000000

C 1.440940000000 2.027949000000 -0.136821000000

C 0.703607000000 3.176977000000 0.027510000000

C -1.245019000000 2.004520000000 0.086063000000

H -1.100824000000 -0.011177000000 0.384778000000

H 1.208809000000 -0.143882000000 -0.494614000000

H 2.522013000000 2.066226000000 -0.245882000000

N 1.218861000000 4.430228000000 0.248005000000

H 2.199134000000 4.563873000000 0.043351000000

H 0.623278000000 5.186888000000 -0.062830000000

N -0.699433000000 3.161978000000 -0.024306000000

O -2.556665000000 1.899859000000 0.174279000000

N -0.592514000000 0.819541000000 0.121193000000

| **Energies (a.u.)** | | -394.361388 |
| --- | --- | --- |
| *E* | 0.096215 | |
| Zero-point correction | -394.265173 | |
| Sum of electronic and  thermal enthalpies | -394.257090 | |
| Sum of electronic and  thermal free energies | -394.296149 | |

**Table S5.** Optimized Cartesian coordinates and energies of the cytosine’s $n\to\pi^{\boldsymbol{*}}$state at the PBE0/6-31+G(d) level of theory in vacuum. Results obtained with SDNTO.

C 0.782302206500 0.793413373250 -0.208572820250

C 1.442307485500 2.026550527250 -0.122263643750

C 0.716954940500 3.176099171750 0.065580595750

C -1.234047926750 2.010118374000 0.141870356000

H -1.146205999250 -0.027898069500 0.065364520000

H 1.253562859000 -0.176703687000 -0.224939590500

H 2.523471660750 2.059894561250 -0.219973134000

N 1.246671888500 4.436314918500 0.211505832500

H 2.175355362250 4.585832085500 -0.156757941000

H 0.595115595750 5.180522250000 0.000090130500

N -0.686563875250 3.169786600500 0.124119746250

O -2.552919483500 1.913364554000 0.234158873000

N -0.600997714750 0.820266339750 0.082500076250

| **Energies (a.u.)** | | -394.360761 |
| --- | --- | --- |
| *E* | 0.095683 | |
| Zero-point correction | -394.265078 | |
| Sum of electronic and  thermal enthalpies | -394.257371 | |
| Sum of electronic and  thermal free energies | -394.295687 | |

**Table S6.** Optimized Cartesian coordinates and energies of the *cis*-(Cl,Cl)[RuCl_2_(NO)(tpy)]^+^ complex ground state at the B3LYP/6-31G(d) level of theory in vacuum.

Ru 3.284023930744 0.089928104448 0.181234953782

Cl 3.107072983269 -0.069590906658 -2.148984774420

Cl 5.660850556346 0.236297927604 -0.037238605488

N 1.280306819801 -0.047955403938 0.138609764793

N 2.775215204012 2.109345901533 -0.071967821351

N 3.052044971335 -1.995048289245 0.187963704044

N 3.486165152605 0.213349344483 1.913792341125

O 3.689353983510 0.297816442839 3.032986575445

C 0.583981331485 1.090722458538 -0.006399476087

C 1.424225067839 2.306898758940 -0.117294582458

C 3.155455582313 4.426369665863 -0.430898288204

H 3.872007907661 5.238598355614 -0.561273658006

C 0.743794813455 -1.278658604757 0.143293657281

C -0.640127755447 -1.398526062943 0.062127668212

H -1.122397012853 -2.375290850257 0.065271682283

C 3.741964782390 -4.266935905519 0.120583961374

H 4.561852441734 -4.985870596577 0.087753792384

C 1.740076950574 -2.375699637187 0.178987541861

C 0.914499620264 3.582487383105 -0.306470152811

H -0.162140820513 3.746421260367 -0.343481333884

C -0.802814833128 1.013292347669 -0.090179382516

H -1.411403385091 1.909414384766 -0.205280637675

C 3.616994078786 3.130988335692 -0.233446679578

H 4.679526387631 2.879038722738 -0.210163586192

C 4.024310101905 -2.906986451258 0.149612372689

H 5.043336415071 -2.514194211061 0.132599007497

C 1.407256678424 -3.721596630391 0.156052733842

H 0.362595221265 -4.031504686648 0.148844479794

C 2.418458712761 -4.676455471541 0.129166227189

H 2.164861627279 -5.738430711012 0.107095246851

C 1.789025430798 4.652986912689 -0.462177933667

H 1.395798186319 5.660191249267 -0.615482638596

C -1.403592033790 -0.240470702625 -0.043199954100

H -2.490740098751 -0.318014434543 -0.109837205405

| **Energies (a.u.)** | | -1888.022754 |
| --- | --- | --- |
| *E* | 0.241113 | |
| Zero-point correction | -1887.781641 | |
| Sum of electronic and  thermal enthalpies | -1887.761400 | |
| Sum of electronic and  thermal free energies | -1887.829792 | |

**Table S7.** Optimized Cartesian coordinates and energies of the *cis*-(Cl,Cl)[RuCl_2_(NO)(tpy)]^+^ complex excited state at the BHandHLYP/6-31G(d) level of theory in vacuum. Results obtained with SDNTO.

Ru 3.278623891000 0.092747978500 0.231612712500

Cl 3.177201135000 -0.082514515500 -2.430581248000

Cl 5.653439907500 0.249832097500 0.183105616500

N 1.274420109000 -0.050326406500 0.107459682000

N 2.762904196000 2.104671887000 -0.079130675500

N 3.039271528500 -1.993036371000 0.180545274000

N 3.340963717500 0.216692210000 2.121348652000

O 4.096881375500 0.320982168000 2.966349398500

C 0.580696253500 1.078964610500 -0.027414998500

C 1.418810610000 2.298143429500 -0.134630726000

C 3.137491183000 4.417758270000 -0.433050419000

H 3.840519056000 5.222653888500 -0.555458727500

C 0.739128387500 -1.270169561000 0.121142848500

C -0.640281528500 -1.396084607000 0.053254615000

H -1.117715844000 -2.359057579000 0.066814830500

C 3.722975466000 -4.261177952500 0.117472542000

H 4.528393086500 -4.973725829500 0.091639849500

C 1.733667905500 -2.370005642500 0.160909146000

C 0.902513210500 3.565303224000 -0.314168878000

H -0.160209504500 3.724983502500 -0.348499875000

C -0.802546306500 1.009761519500 -0.098798685000

H -1.404577547500 1.894273022000 -0.201963803000

C 3.597535380500 3.126004197500 -0.238918569000

H 4.645765390000 2.881829713500 -0.203619776500

C 4.004381498000 -2.905420076000 0.143758488500

H 5.010168590500 -2.520673696000 0.139612395000

C 1.393099746500 -3.707275811000 0.146398583500

H 0.361629582000 -4.010763059000 0.141147183000

C 2.400765376000 -4.663354117000 0.128219836500

H 2.147456230500 -5.709931667500 0.114620099000

C 1.773317209500 4.637621509000 -0.461277325000

H 1.382381488500 5.630811844500 -0.604159591000

C -1.402009138500 -0.240455605000 -0.044779283500

H -2.474849641000 -0.316088575500 -0.096577172000

| **Energies (a.u.)** | | -1885.774417 |
| --- | --- | --- |
| *E* | 0.251573 | |
| Zero-point correction | -1885.502406 | |
| Sum of electronic and  thermal enthalpies | -1885.571944 | |
| Sum of electronic and  thermal free energies | 0.251573 | |

**Figure S1.** Superposition of the optimized structure with a standard algorithm (red) and SDNTO (blue).

| $\pi\to\pi^{\boldsymbol{*}}$ | $n\to\pi^{\boldsymbol{*}}$ |
| --- | --- |


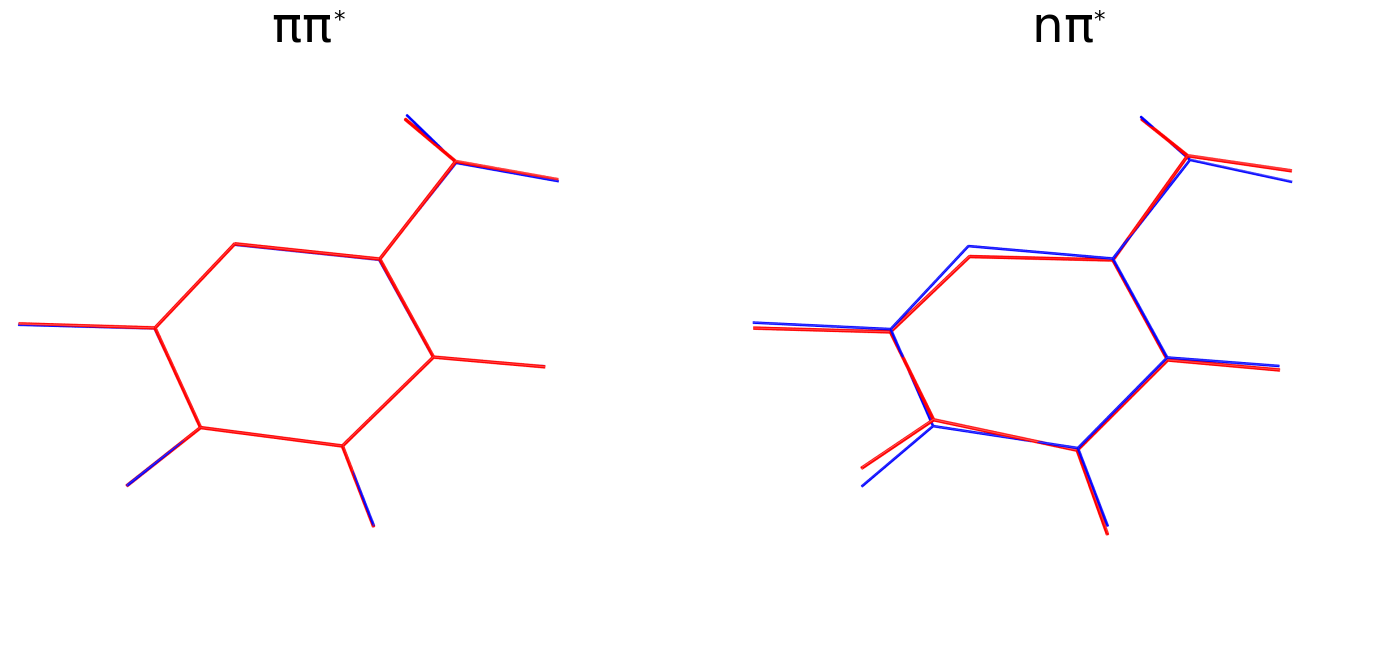


N.B. An additional steepest decent optimization of the $n\to\pi^{\boldsymbol{*}}$ SDNTO structure affords a practically identical geometry to the one obtained with a standard algorithm. Comparing both structures (see structures below) the computed RMSD is only 0.008 Å and the energy difference is 0.002 kcal/mol.

| $n\to\pi^{\boldsymbol{*}}$ |
| --- |


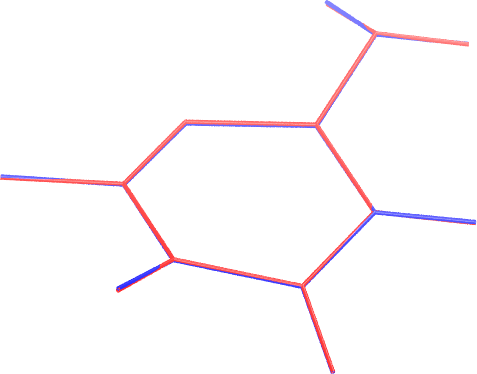


**Table S8.** Displacement of each state crossing during the *cis*-(Cl,Cl)[RuCl_2_(NO)(tpy)]^+^ SDNTO optimization reported in Figure 4, using initially *α* = 0.2 Bohr^2^ / Hartree and then *α* = 0.9 Bohr^2^/ Hartree for the last optimization steps.

|  |  |  | **NTOs** | |
| --- | --- | --- | --- | --- |
| ***α */**  **Bohr^2^ · Hartree^-1^** | **Displacement** **length** ⭢  **/ Bohr** | **State Crossing** | **RS Hole (*n*)** | **RS Hole (*n + 1*)** |
| 0.2 | 0.008 | S_9_ – S_10_ | 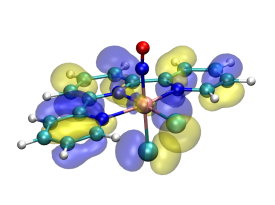 | 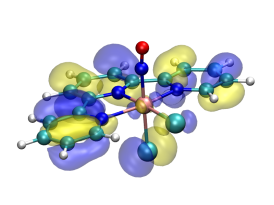 |
| 0.2 | 0.007 | S_10_ – S_9_ | 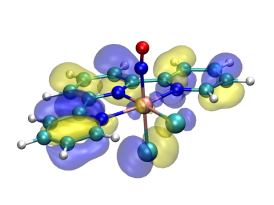 | 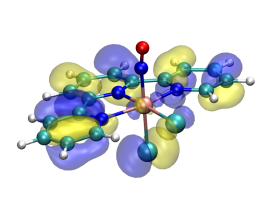 |
| 0.2 | 0.006 | S_9_ – S_8_ | 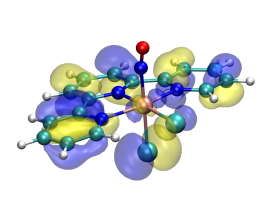 | 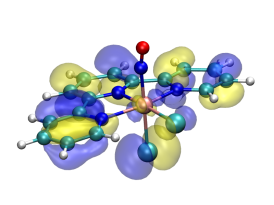 |
| 0.9 | 0.010 | S_8_ – S_7_ | 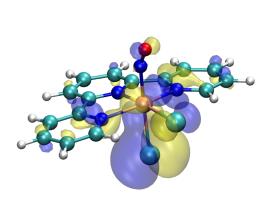 | 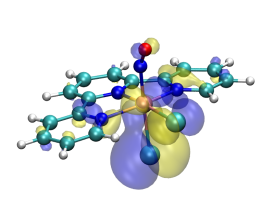 |
| 0.9 | 0.008 | S_7_ – S_6_ | 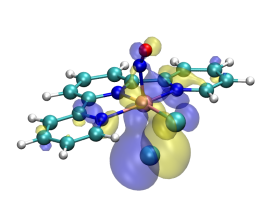 | 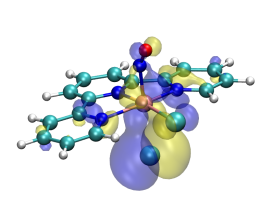 |

|  |  |  | **NTOs** | |
| --- | --- | --- | --- | --- |
| ***α */**  **Bohr^2^ · Hartree^-1^** | **Displacement** **length** ⭢  **/ Bohr** | **State Crossing** | **RS Particle (*n*)** | **RS Particle (*n + 1*)** |
| 0.2 | 0.008 | S_9_ – S_10_ | 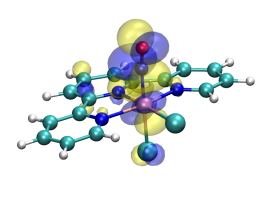 | 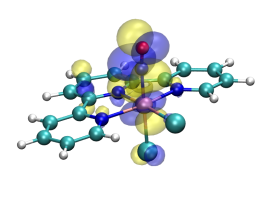 |
| 0.2 | 0.007 | S_10_ – S_9_ | 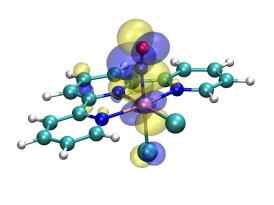 | 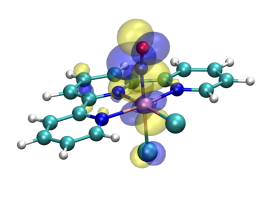 |
| 0.2 | 0.006 | S_9_ – S_8_ | 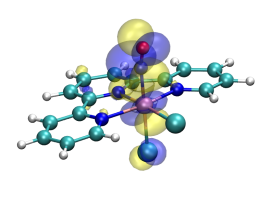 | 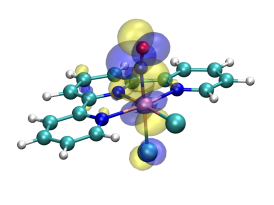 |
| 0.9 | 0.010 | S_8_ – S_7_ | 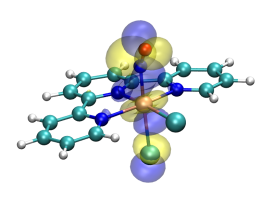 | 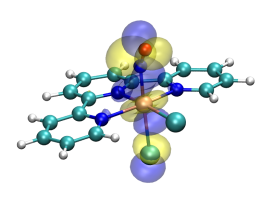 |
| 0.9 | 0.008 | S_7_ – S_6_ | 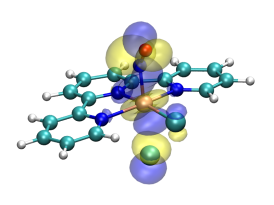 | 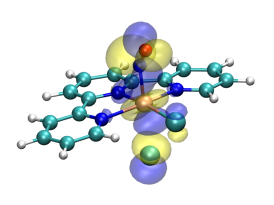 |

**Table S9.** Different displacements of each state crossing during two additional *cis*-(Cl,Cl)[RuCl_2_(NO)(tpy)]^+^ SDNTO optimizations, using *α* = 0.8 Bohr^2^ / Hartree for one and *α* = 0.9 Bohr^2^ / Hartree for the other.

|  |  |  | **NTOs** | |
| --- | --- | --- | --- | --- |
| ***α */**  **Bohr^2^ · Hartree^-1^** | **Displacement** **length** ⭢  **/ Bohr** | **State Crossing** | **RS Hole (*n*)** | **RS Hole (*n + 1*)** |
| 0.8 | 0.029 | S_9_ – S_10_ | 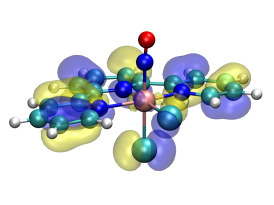 | 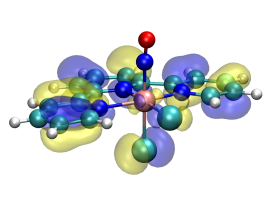 |
| 0.9 | 0.110 | S_9_ – S_10_ | 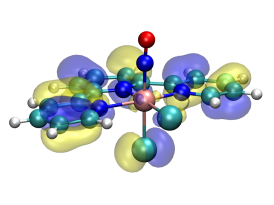 | 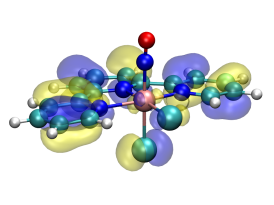 |
| 0.8 | 0.026 | S_10_ – S_9_ | 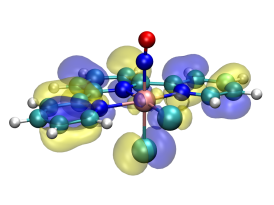 | 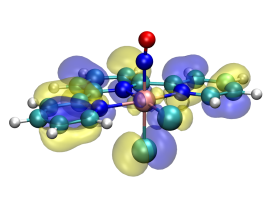 |
| 0.9 | 0.048 | S_10_ – S_9_ | 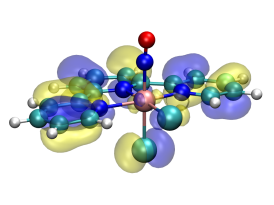 | 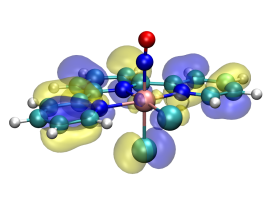 |
| 0.8 | 0.025 | S_9_ – S_8_ | 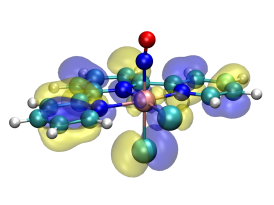 | 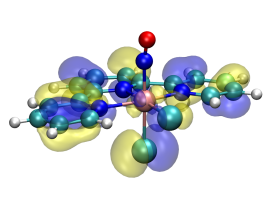 |
| 0.9 | 0.053 | S_9_ – S_8_ | 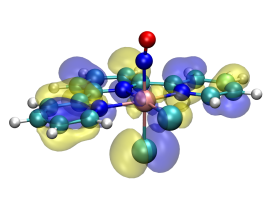 | 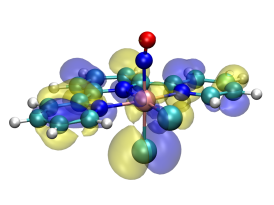 |
| 0.8 | 0.022 | S_8_ – S_7_ | 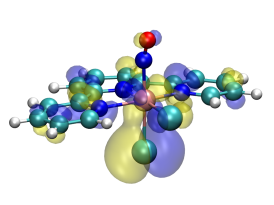 | 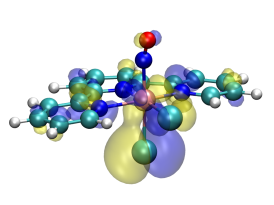 |
| 0.9 | 0.073 | S_8_ – S_7_ | 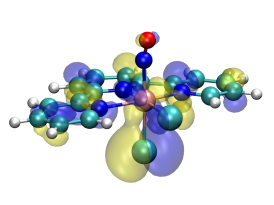 | 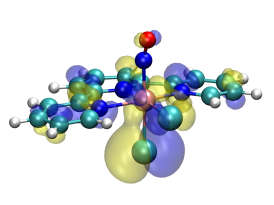 |
| 0.8 | 0.019 | S_7_ – S_6_ | 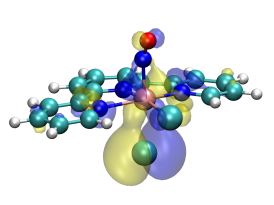 | 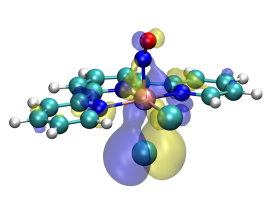 |
| 0.9 | 0.037 | S_7_ – S_6_ | 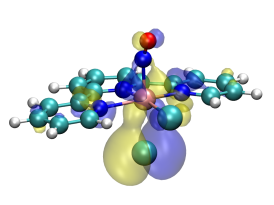 | 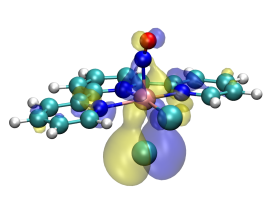 |

|  |  |  | **NTOs** | |
| --- | --- | --- | --- | --- |
| ***α */**  **Bohr^2^ · Hartree^-1^** | **Displacement** **length** ⭢  **/ Bohr** | **State Crossing** | **RS Particle (*n*)** | **RS Particle (*n + 1*)** |
| 0.8 | 0.029 | S_9_ – S_10_ | 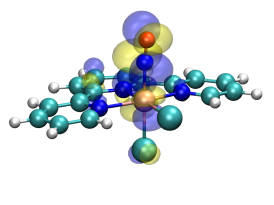 | 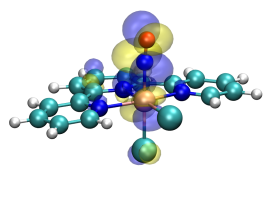 |
| 0.9 | 0.110 | S_9_ – S_10_ | 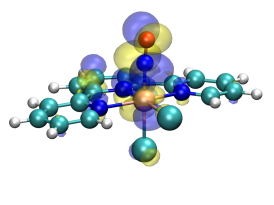 | 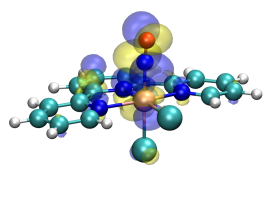 |
| 0.8 | 0.026 | S_10_ – S_9_ | 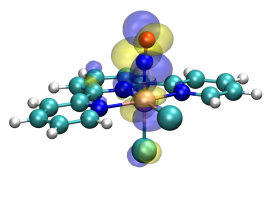 | 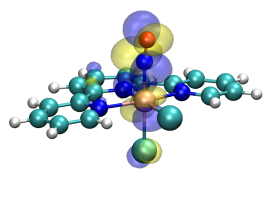 |
| 0.9 | 0.048 | S_10_ – S_9_ | 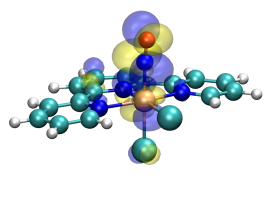 | 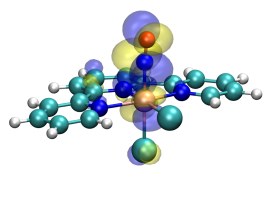 |
| 0.8 | 0.025 | S_9_ – S_8_ | 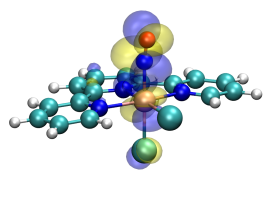 | 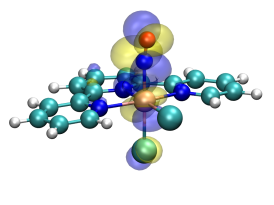 |
| 0.9 | 0.053 | S_9_ – S_8_ | 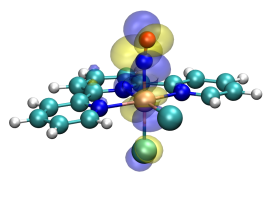 | 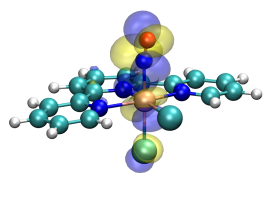 |
| 0.8 | 0.022 | S_8_ – S_7_ | 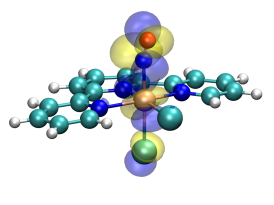 | 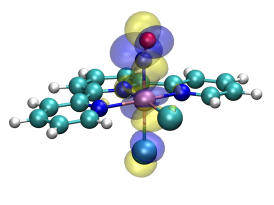 |
| 0.9 | 0.073 | S_8_ – S_7_ | 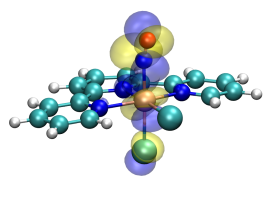 | 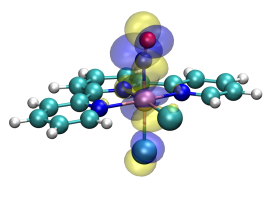 |
| 0.8 | 0.019 | S_7_ – S_6_ | 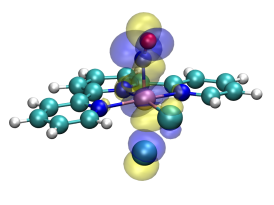 | 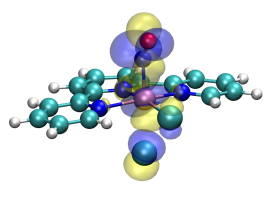 |
| 0.9 | 0.037 | S_7_ – S_6_ | 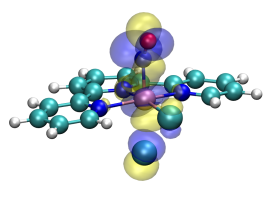 | 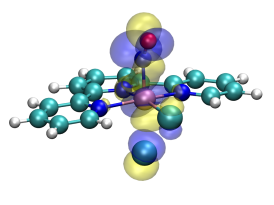 |

**Table S10.** Comparing the standard and the NTO-based state-traking approaches.

The following analysis has been performed using (in both cases) the same structures taken from the standard excited state optimization algorithm. Thus, the step-size for the comparison of both state-tracking algorithms is strictly the same.

| **Standard State-Tracking Approach** | | | **NTO-Based State-Tracking Approach** | | |
| --- | --- | --- | --- | --- | --- |
|  | **NTOs** | |  | **NTOs** | |
| **State Crossing** | **RS Hole (*n*)** | **RS Hole (*n + 1*)** | **State**  **Crossing** | **RS Hole (*n*)** | **RS Hole (*n + 1*)** |
| S_9_ – S_10_ | 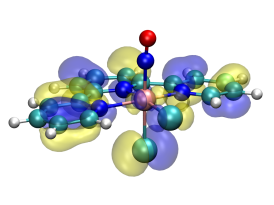 | 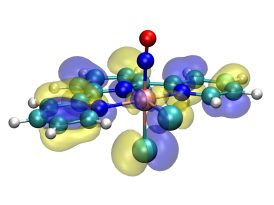 | S_9_ – S_10_ | 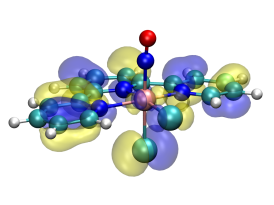 | 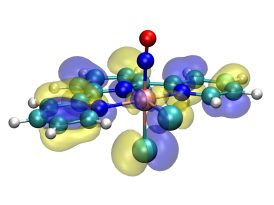 |
| S_10_ – S_9_ | 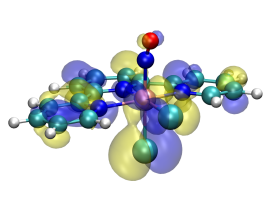 | 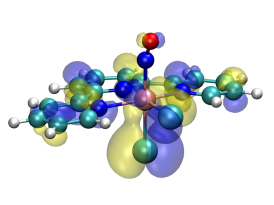 | S_10_ – S_9_ | 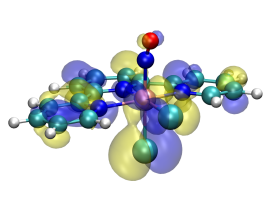 | 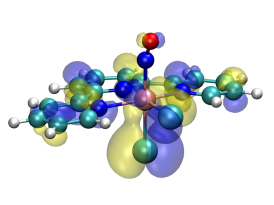 |
| *^a^*S_9_ – S_9_ | 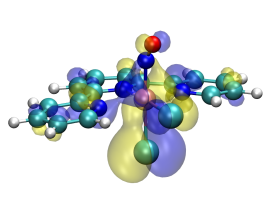 | 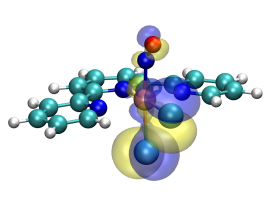 | \| S_9_ – S_7_ \| \| --- \| | 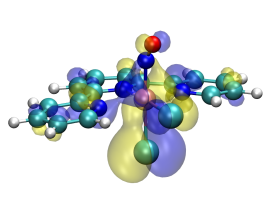 | 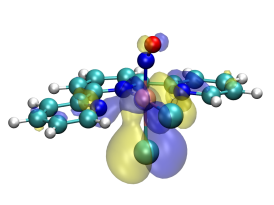 |

| **Standard State-Tracking Approach** | | | **NTO-Based State-Tracking Approach** | | |
| --- | --- | --- | --- | --- | --- |
|  | **NTOs** | |  | **NTOs** | |
| **State Crossing** | **RS Particle (*n*)** | **RS Particle (*n + 1*)** | **State**  **Crossing** | **RS Particle (*n*)** | **RS Particle (*n + 1*)** |
| S_9_ – S_10_ | 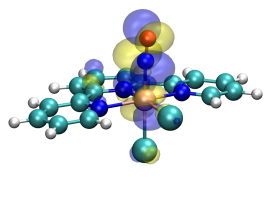 | 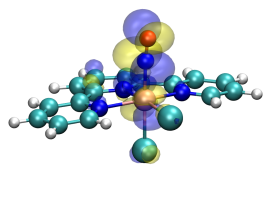 | S_9_ – S_10_ | 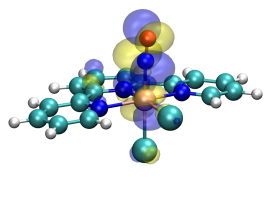 | 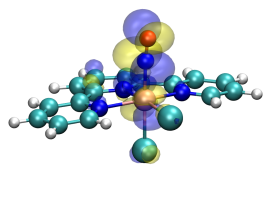 |
| S_10_ – S_9_ | 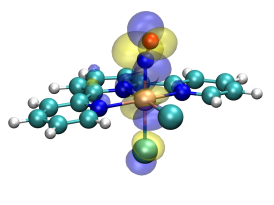 | 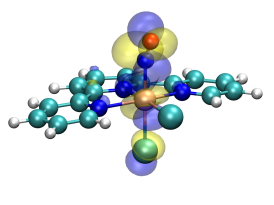 | S_10_ – S_9_ | 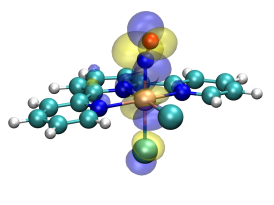 | 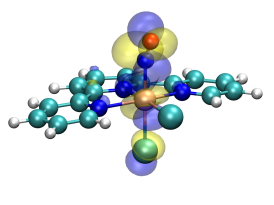 |
| *^a^*S_9_ – S_9_ | 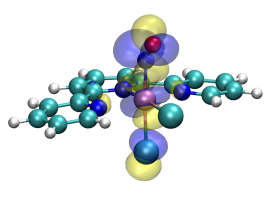 | 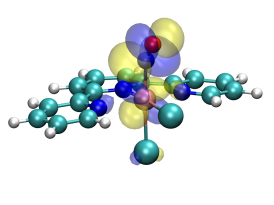 | \| S_9_ – S_7_ \| \| --- \| | 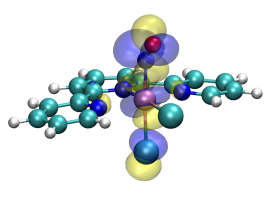 | 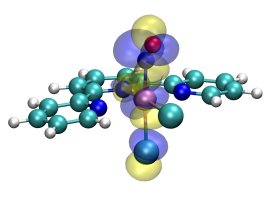 |

*^a^*This step is not a crossing in the standard optimization (S_9_ – S_9_). At this point, the program fails to follow the electronic nature of the excited state of interest (diabatic state), as it can be seen by the nature of the NTOs involved in the consecutive steps. However, as it can clearly be seen, the NTO’s overlap-based state-tracking is able to correctly follow the excited state of interest not only in the steps where the standard approach succeeds but also in this step where the standard approach fails.

**Table S11.** NTOs (hole) of the diabatic states from the *cis*-(Cl,Cl)[RuCl_2_(NO)(tpy)]^+^ SDNTO optimization reported in Figure 4 and 5, from step 479 to step 489.


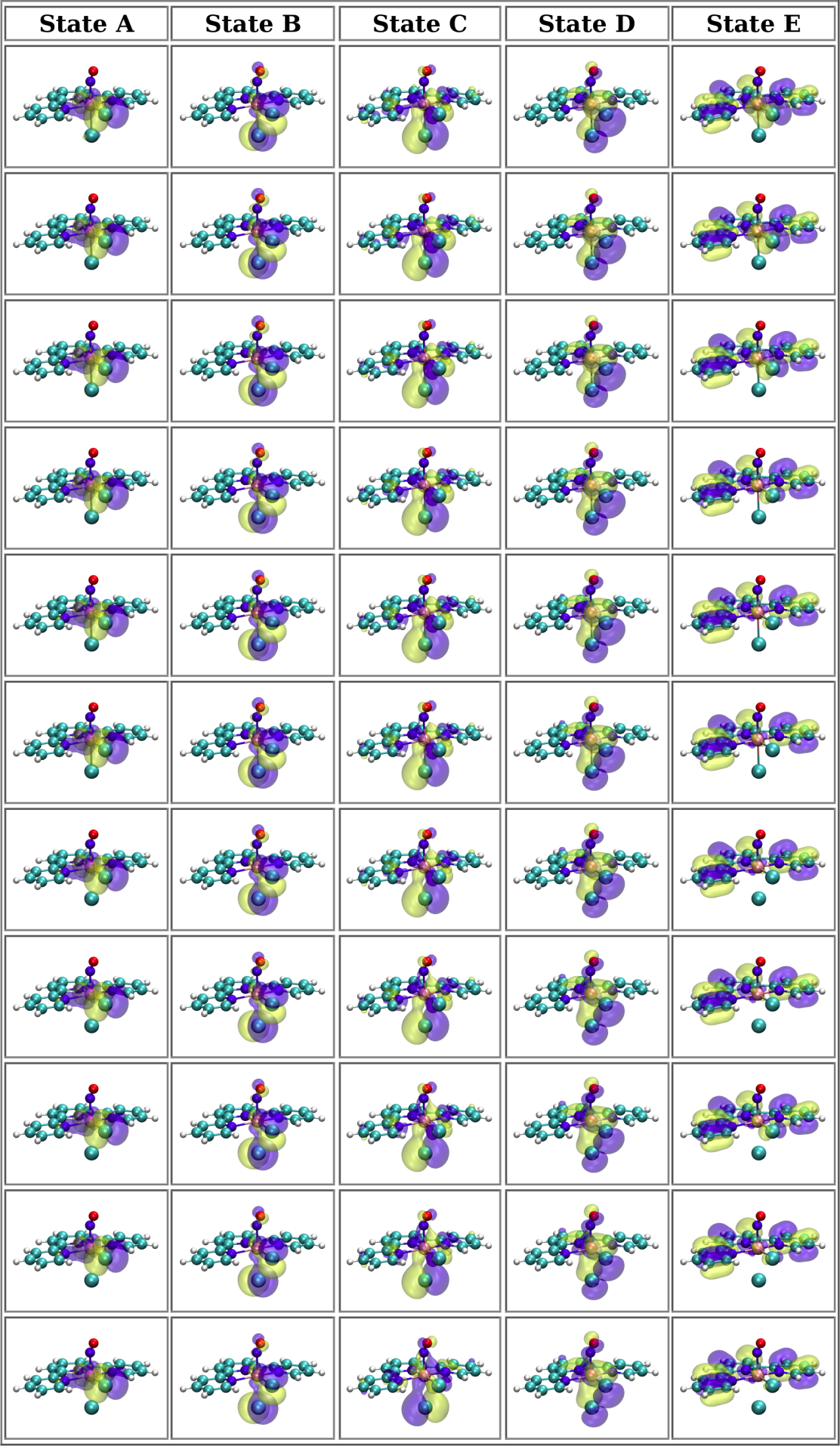


**Table S12.** NTOs (particle) of the diabatic states from the *cis*-(Cl,Cl)[RuCl_2_(NO)(tpy)]^+^ SDNTO optimization reported in Figure 4 and 5, from step 479 to step 489.


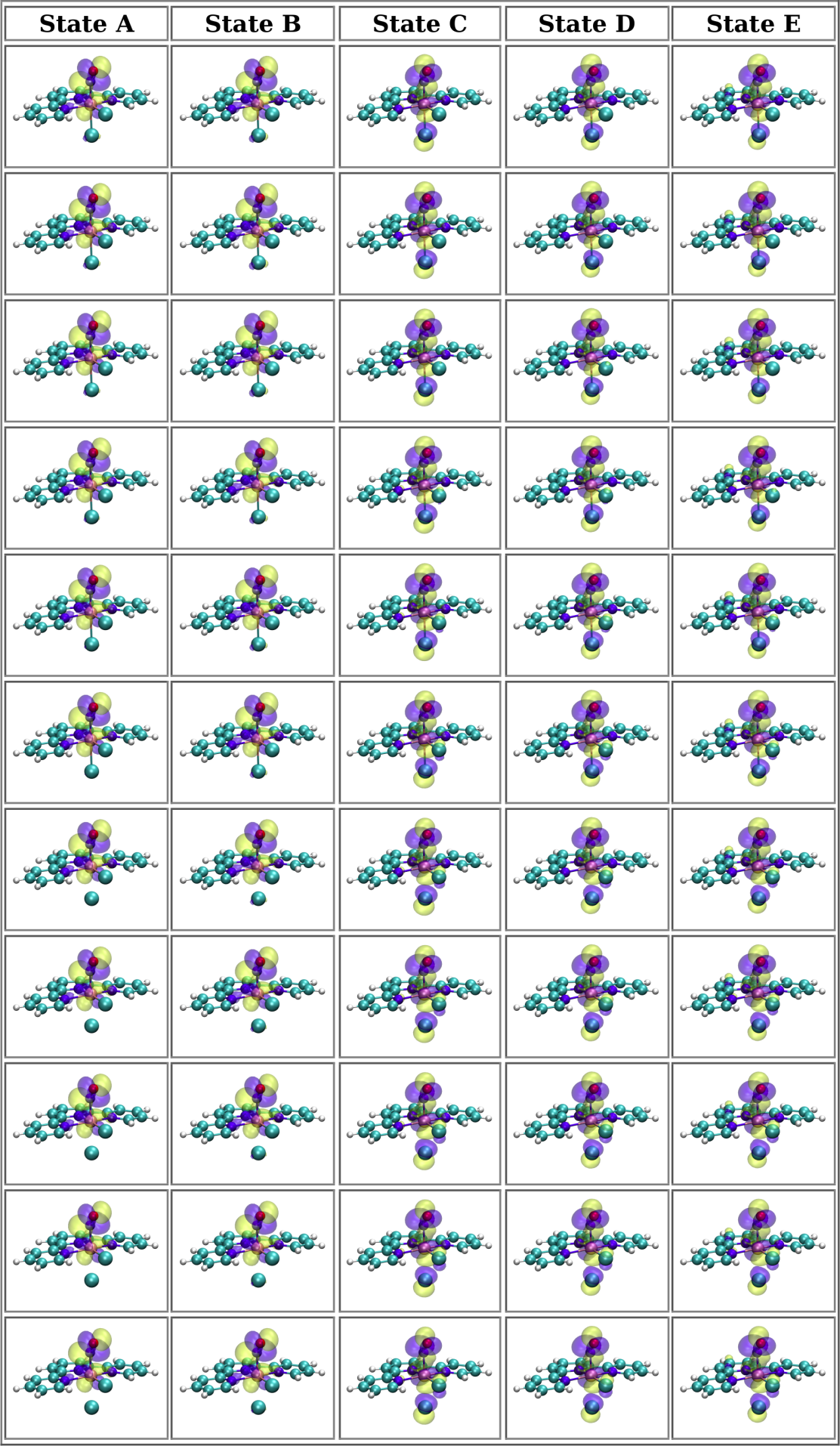

Supplement: Supplementary file 2 — Appendix S2: Supporting Information [file JCC-40-1420-s002.docx]
